# Phase Angle as a Marker of Physical Function in Non-Sarcopenic Rheumatoid Arthritis

**DOI:** 10.3390/medicina60030355

**Published:** 2024-02-21

**Authors:** Hae-Yeon Park, Jong In Lee, Yune-Jung Park, Seong Hoon Lim

**Affiliations:** 1 Department of Rehabilitation Medicine, Bucheon St. Mary’s Hospital, College of Medicine, The Catholic University of Korea, Seoul 06591, Republic of Korea; 2 Department of Rehabilitation Medicine, Seoul St. Mary’s Hospital, College of Medicine, The Catholic University of Korea, Seoul 06591, Republic of Korea; 3 Division of Rheumatology, Department of Internal Medicine, St. Vincent’s Hospital, College of Medicine, The Catholic University of Korea, Seoul 06591, Republic of Korea

**Keywords:** rheumatoid arthritis, RA, strengthening exercise, arthritis, exercise, mental heath

## Abstract

*Background*: Rheumatoid arthritis (RA) is a chronic autoimmune inflammatory disease. Most patients with RA face a barrier to participation in social activities or exercise due to joint pain, despite the beneficial effects of exercise and physical activity. Thus, RA may be a risk factor for sarcopenia in the clinical field. Bioelectrical impedance analysis-derived phase angle (PhA) reflects cellular health and is correlated with the prognosis of various diseases. However, its association with physical function in non-sarcopenic RA female patients remains unclear. We evaluated the association between PhA values and various physical function measures in female patients with non-sarcopenic RA. *Methods*: Thirty-five participants with RA were screened. One met the criteria for sarcopenia. Finally, 34 patients with non-sarcopenic RA were enrolled. This cross-sectional retrospective study evaluated upper- and lower-extremity strengths, cross-sectional area of rectus femoris, 6 min walking test, Borg scale score, sit-to-stand test, and physical function and mental health from 36-Item Short Form Health Survey scores. *Results*: In total, 34 female participants (mean age = 49.74 ± 8.15 years) were enrolled. In non-sarcopenic RA patients, PhA was significantly correlated with BMI and ASM/(height)^2^. Multicollinearity was not detected among the independent variables (VIF < 5). The final multivariable regression model identified ASM/height^2^ as a significant predictor of PhA among non-sarcopenic RA patients. *Conclusion*: Multivariable linear regression analyses identified appendicular skeletal muscle mass as a significant predictor of PhA. Bioelectrical impedance analysis-derived PhA is a valuable guidance tool for RA management. PhA can be a useful clinical biomarker of muscle status in non-sarcopenic RA patients.

## 1. Introduction

Rheumatoid arthritis (RA), a chronic, systemic, autoimmune inflammatory disease, primarily affects the joints. Although exercise and physical activity are beneficial for RA patients [1,2], comorbid sarcopenia increases the risks of falls, fractures, and joint inflammation [3]. Sarcopenia is a progressive and generalized skeletal muscle disorder that involves the accelerated loss of muscle strength and mass [4]. Most patients with RA or other arthritis face barriers to participation in social activities or exercise due to joint pain, despite the beneficial effects of exercise and physical activity [5,6]. Our previous studies demonstrated the effectiveness of systemic strength training, including of the upper and lower extremities, in improving muscle strength and quality of life among RA patients [2].

For the diagnosis of sarcopenia, several tools were used, as follows: assessing grip strength, determining appendicular skeletal muscle mass divided by height^2^, assessing gait speed, the timed up-and-go test, and, recently, an ultrasound of skeletal muscles [1,4,7]. Among the several diagnostic tools, bioimpedance analysis (BIA), a safe, non-invasive, electrical-current-based tool, evaluates body composition, including fat-free mass, total body water, and extracellular and intracellular water [8]. BIA is commonly used to estimate nutritional status and muscle mass; it has proven valuable for sarcopenia diagnosis in older adults [1]. The BIA-derived phase angle (PhA) reflects cellular health and is correlated with the prognosis of various diseases [9,10,11]. Recent studies have emphasized its potential for diagnosing sarcopenia [12]. A low PhA is associated with diminished muscle strength [13], and is prevalent in sarcopenic patients [14,15,16]. Additionally, a low PhA may predict falls in older adults [17].

Phase angle (PhA) is a parameter derived from BIA, which serves as an indicator of cell membrane integrity and cellular health [9]. Previous studies have demonstrated a significant association between PhA and the prognosis of diverse pathologies, including cancer and cardiovascular diseases, where a decreased PhA correlates with worse prognoses [9,10,11]. Additionally, PhA has been validated as a useful biomarker for the assessment of nutritional status, which is able to predict disease-related malnutrition [9,18]. Recent studies have also highlighted its role in identifying sarcopenia, noting that a lower PhA is linked to diminished muscle strength and/or functionality [12,13,14,15,16]. In particular, lower PhA was associated with decreased grip strength value in elderly patients with prostate cancer [12], those experiencing acute stroke [19], and those with chronic kidney disease [20] Moreover, a low PhA may predict increased fall risk in geriatric populations [17].

Based on these findings, indicating that low PhA is typically associated with poor outcomes, we hypothesized that the BIA-derived PhA is associated with physical functions in non-sarcopenic RA patients. We evaluated the associations between PhA values and various physical function measures in female patients with non-sarcopenic RA.

## 2. Methods

### 2.1. Study Design and Participants

This cross-sectional, retrospective study enrolled female adults (age > 18 years-old) with a stable state of rheumatoid arthritis (no changes in the use of disease-modifying anti-rheumatic drugs or steroid for the last 3 months) were included in the study. The exclusion criteria included those unable to weight-bear with lower limbs, those with serious medical comorbidities or an unstable heart condition, and those diagnosed with sarcopenia. One rheumatologist performed a screening of disease activity, based on clinical and laboratory data. Stable disease, defined as a change in Disease Activity Score (DAS28), was ≤3.2 (low current disease activity) and the difference in DAS28 scores between baseline and the last measurement was ≤1.2 [2,21]. The patients analyzed in this study are a secondary analysis, conducted using data from patients who participated in a previous RCT [2].

The Ethics Committee of the Catholic University of Korea approved the study protocol (approval number: VC18FESI0049). Informed consent was obtained from all participants. 

### 2.2. Evaluation of Sarcopenia

The 2019 consensus definition of the Asian Working Group for Sarcopenia (AWGS) was used to identify individuals with sarcopenia, characterized by low appendicular skeletal muscle mass (ASM), along with low muscle strength or poor physical performance [1]. Notably, low ASM was defined as ASM/(height)^2^ < 5.7 kg/m^2^, low muscle strength as handgrip strength < 18 kg for women, and poor physical performance as gait speed < 1.0 m/s. 

ASM, which is the sum of the upper and lower extremities muscle mass, was obtained from the bioelectrical impedance analysis (BIA, InBody S10; Biospace Co., Ltd., Seoul, Republic of Korea) performed at enrollment. With the supine position, a total of eight electrodes were attached on both wrists and ankles to evaluate BIA. Along with ASM, phase angle (PhA) was also calculated from the built-in equation from Inbody S10 using resistance (R) and reactance (Xc) [9]. 

### 2.3. Functional Assessment

Bilateral handgrip strength was assessed using an electronic handgrip dynamometer (KH-100, Kyung In, Seoul, Republic of Korea). Participants were seated with their shoulders adducted, elbows flexed at 90°, and arms in neutral positions. Grip strength was measured as the maximum force exerted during a single isometric contraction. Lower-extremity strength was evaluated using a handheld dynamometer (JTech Medical, Salt Lake City, UT, USA). Participants were seated with back support and knees flexed at 90°; isometric quadriceps contractions were evaluated. Three measurements were performed to assess upper- and lower-extremity strengths, and the mean values were used for analysis. 

Quadriceps’ strength and the cross-sectional area of the rectus femoris were assessed via ultrasound (Accuvix XQ, Samsung Medison, Seoul, Republic of Korea) using an 8–13 MHz linear array transducer. With the knee fully extended, the probe was placed perpendicular to the long axis of the thigh, three-quarters of the distance from the anterior superior iliac spine to the superior border of the patella [7].

Gait speed was evaluated using a 6 min walk test (6MWT). Participants were instructed to walk a 20 m course, and the maximum distance covered in 6 min was recorded. Subsequently, perceived exertion was assessed using a Borg scale score (0–10). Endurance was evaluated using a 30 s sit-to-stand test, where participants repeatedly sat down and stood up from a chair without armrests for 30 s [22].

Quality of life was evaluated using the Korean version of the 36-item Short-Form Health Survey (SF-36) [23]. Among the eight subscales, physical function and mental health domains were assessed in the analysis. 

### 2.4. Statistical Analysis

Statistical analyses were performed using R software, version 4.1.2 (R Foundation for Statistical Computing, Vienna, Austria). Sample size estimation was based on the methodology outlined in a previous study [2]. Univariable linear regression analyses were utilized to identify factors associated with PhA. Subsequently, multivariable linear regression analyses were used to assess the relationships of PhA and multiple variables. Collinearity was assessed using the variance inflation factor (VIF), where a threshold of VIF > 10 indicated potential problems. *p*-values < 0.05 were considered statistically significant. 

## 3. Results

### 3.1. Baseline Characteristics of the Participants

Among the 35 participants from our previous study, only 1 met the criteria for sarcopenic RA. Therefore, the final analyses included 34 non-sarcopenic RA patients. Although three participants demonstrated ASM/(height)^2^ < 5.7 kg/m^2^, they did not exhibit low muscle strength or a poor physical performance, which are characteristic of sarcopenia. Table 1 and Table 2 present the baseline clinical characteristics and disease characteristics for RA in all subjects.

### 3.2. Factors Associated with the Phase Angle

In non-sarcopenic RA patients, PhA was significantly correlated with BMI and ASM/(height)^2^. Multicollinearity was not detected among the independent variables (VIF < 5). The final multivariable regression model identified ASM/height^2^ as a significant predictor of PhA among non-sarcopenic RA patients (Table 3). No significant associations were observed with other functional outcome parameters. 

## 4. Discussion

The BIA-derived PhA holds promise in clinical settings due to its ease of measurement and reliable representation of nutritional status and muscle mass in sarcopenic and cancer patients [12,16,24,25]. Therefore, we investigated the value of BIA-derived PhA in non-sarcopenic RA patients. Our findings revealed a significant association between PhA and BMI and ASM/(height)^2^, indicating its potential to reflect body muscle mass. However, unlike previous studies, a direct correlation was not observed between BIA-derived PhA values and grip strength, low muscle strength, or impaired physical performance in our non-sarcopenic RA patients [10,12,15,16]. These findings suggest that the clinical applications of BIA-derived PhA for evaluating physical performance in non-sarcopenic patients are limited.

Sarcopenia is an age-related syndrome with known risk factors, including low protein intake, gastrointestinal issues, immobility, reduced physical activity, and underlying diseases [4]. The risk factors of sarcopenia were known to be age, a longer disease duration, malnutrition, DAS28, C-reactive protein, menopause, and rheumatoid factor seropositivity [26,27,28]. According to the AWGS (2019), the prevalences of sarcopenia are 6.7–21.8% and 13.7–23.4% in male and female community-dwelling older adults, respectively [29,30,31,32]. Notably, RA patients exhibit a significantly higher prevalence of sarcopenia, reaching 25.0% in women [27]. Muscle strength and muscle mass are the key markers of sarcopenia [3]. This definition and prevalence emphasizes the potential of BIA as an accessible and easy-to-use tool for the clinical evaluation of RA patients. However, our findings suggest that BIA-derived PhA is not a reliable indicator of physical performance in non-sarcopenic RA patients. Consistent with the results of previous studies, the BIA-derived PhA appears more suitable for assessing muscle mass relative to body mass, rather than physical performance, in RA patients.

A previous study demonstrated that the BIA is a simple and reproducible method for assessing the electrical properties of the human body, as well as muscle structure and strength, which can be used throughout the nutrition care process [14,16,33]. Another study of our team showed that a lower PhA was associated with older age and lower maximal grip strength value in older patients with prostate cancer [12]. Moreover, the current results revealed that PhA may represent muscle mass relative to body mass in non-sarcopenic RA patients. The PhA might be better utilized as a clinical biomarker for clinically reflecting muscle mass relative to body mass, not only in sarcopenic patients but also in non-sarcopenic patients.

A previous study demonstrated that the BIA is a simple and reproducible method for assessing the electrical properties of the human body, as well as muscle structure and strength, which could be used throughout the nutrition care process [14,16,33]. Moreover, the current results revealed that PhA may represent muscle mass relative to body mass in non-sarcopenic RA patients. This result is similar to previous studies that found PhA to be a reliable indicator of muscle mass and function across various populations, including those with chronic illnesses, various types of cancer, and stroke [12,16,18,20,25,34]. The correlation between PhA and muscle mass relative to body mass in non-sarcopenic RA patients highlights its potential as a non-invasive tool for evaluating muscle mass in this specific patient group. Clinically, PhA might be better utilized as a biomarker for reflecting muscle mass relative to body mass, not only in sarcopenic patients but also in non-sarcopenic patients.

Our study has several limitations. First, it utilized a single-center, cross-sectional, retrospective design, and a relatively small sample size. Therefore, we enrolled female and ethnically homogenous patients, which reduced participant heterogeneity. Second, unlike previous studies, we did not identify an association between the PhA and nutritional status [10,11], Nevertheless, our study demonstrated significant associations between PhA and BMI and ASM/(height)^2^ in non-sarcopenic RA patients. Future prospective studies with larger samples are needed to explore the potential benefits of PhA in non-sarcopenic RA patients.

## 5. Conclusions

Considering the morbidity and burden associated with RA, BIA-derived PhA has merit for evaluating the status of skeletal muscle for RA management. The PhA could be a useful clinical biomarker of appendicular skeletal muscle mass in non-sarcopenic RA patients. 

## Figures and Tables

**Table 1:** Baseline participant characteristics (*n* = 34).

	Total (*n* = 34)
Age (years)	49.74 ± 8.15
BMI (kg/m^2^)	24.11 ± 3.66
ASM/(height)^2^ (kg/m^2^)	6.44 ± 0.66
Phase angle (degrees)	5.18 ± 0.57
Disease duration (years)	5.41 ± 7.01
UE strength (Rt) (kg)	21.54 ± 5.08
UE strength (Lt) (kg)	21.05 ± 3.69
LE strength (Rt) (kg)	24.95 ± 3.04
LE strength (Lt) (kg)	24.47 ± 2.63
CSA-RF (Rt)	1.23 ± 0.62
CSA-RF (Lt)	1.16 ± 0.60
6 min walk test (m)	500.03 ± 48.51
30 s sit-to-stand	16.68 ± 3.32
Borg score after 6MWT	2.12 ± 0.98
Physical function (SF-36)	72.65 ± 14.31
Mental health (SF-36)	56.18 ± 8.81

Values are given as means ± standard deviations. BMI, body mass index; UE, upper extremity; LE, lower extremity; CSA-RF (cross-sectional area of the rectus femoris); 6MWT, 6 min walk test; SF-36, 36-Item Short-Form Health Survey; ASM, appendicular skeletal muscle mass.

**Table 2:** Disease characteristics for RA (*n* = 34).

	Total (*n* = 34)
RA-related characteristics	
Disease duration, years	5.41 ± 7.01
Rheumatoid factor positivity	25 (73.5%)
Anti-CCP antibody positivity	27 (79.4%)
DAS28-ESR	2.88 ± 1.00
DAS28-CRP	2.29 ± 0.79
RA medication	
cDMARD monotherapy	9 (26.5%)
cDMARD combination	20 (58.8%)
Biologics	5 (14.7%)

Values are given as means ± standard deviations. ESR, erythrocyte sedimentation rate; CRP, C-reactive protein; DAS, disease activity score; DMARD, disease-modifying antirheumatic drug. The number 28 refers to the 28 joints that are examined in this assessment.

**Table 3:** Factors associated with phase angle in non-sarcopenic rheumatoid arthritis patients.

	Univariable		Multivariable	
	Correlation Coefficient	*p*-Value	Regression Coefficient	*p*-Value
Age (yrs)	−0.233	0.184		
BMI (kg/m^2^)	**0.349**	0.043 *	−0.022	0.536
ASM/(Height)^2^ (kg/m^2^)	**0.545**	<0.001 *	**0.561**	0.007 *
Disease duration (yrs)	0.003	0.986		
UE strength (Rt) (kg)	0.169	0.339		
UE strength (Lt) (kg)	0.136	0.442		
LE strength (Rt) (kg)	0.322	0.063		
LE strength (Lt) (kg)	0.053	0.768		
CSA-RF (Rt)	0.134	0.451		
CSA-RF (Lt)	0.289	0.097		
6 min walk test (m)	0.022	0.903		
30 s sit-to-stand	−0.035	0.844		
Borg after 6MWT	−0.17	0.336		
Physical function (SF-36)	0.026	0.883		
Mental health (SF-36)	−0.049	0.784		

BMI, body mass index; UE, upper extremity; LE, lower extremity; CSA-RF (cross-sectional area of the rectus femoris); 6MWT, 6 min walk test; SF-36, 36-Item Short Form Health Survey, * *p* < 0.05.

## Data Availability

Dataset available on request from the authors.

## Abbreviations

RA, rheumatoid arthritis; CSA-RF, cross-sectional area of the rectus femoris; 6MWT, 6 min walk test; SF-36, 36-Item Short-Form Health Survey.
